# Artificial intelligence based on ultrasound for initial diagnosis of malignant ovarian cancer: a systematic review and meta-analysis

**DOI:** 10.3389/fonc.2025.1626286

**Published:** 2025-12-01

**Authors:** Rong Li, Jiehua Lei, Xiaomei Tang, Shiying Zheng, Jiajia Qu, Yueyue Xu, Hongyu Zheng

**Affiliations:** Departments of Ultrasound, The People’s Hospital of Guangxi Zhuang Autonomous Region, Nanning, Guangxi, China

**Keywords:** artificial intelligence, ovarian neoplasms, ultrasonography, diagnosis, meta-analysis

## Abstract

**Purpose:**

This meta-analysis aimed to evaluate the diagnostic performance of artificial intelligence (AI) in ultrasound imaging for the initial diagnosis of malignant ovarian cancer, comparing its performance to that of sonographers.

**Methods:**

A systematic literature search was conducted in PubMed, Web of Science, Embase, and the Cochrane Library up to February 2025. Inclusion criteria targeted studies employing AI algorithms to analyze ultrasound images in patients with suspected ovarian cancer, using pathology as the reference standard. Bivariate random-effects models were utilized to aggregate sensitivity, specificity, and area under the curve (AUC). The methodological quality of the included studies was assessed using a modified version of the Quality Assessment of Diagnostic Accuracy Studies-2 (QUADAS-2) tool.

**Results:**

Eighteen studies encompassing a total of 22,697 total patients/images/lesions were analyzed. AI demonstrated a sensitivity of 0.95 (95% CI: 0.88-0.98) and specificity of 0.95 (95% CI: 0.89-0.98) in internal validation sets, yielding an AUC of 0.98. In external validation, sensitivity was 0.78 (95% CI: 0.56-0.91) and specificity was 0.88 (95% CI: 0.76-0.95), with an AUC of 0.91. In comparison, sonographers exhibited a sensitivity of 0.83 (95% CI: 0.62-0.94), specificity of 0.84 (95% CI: 0.79-0.88), and an AUC of 0.87. These results indicate that ultrasound-based AI significantly outperforms sonographer diagnostics. Meta-regression analysis indicated that the heterogeneity was primarily attributed to the analysis method (image-based vs. patient-based, specificity *P* = 0.01).

**Conclusions:**

AI based on ultrasound diagnosis demonstrates excellent performance for malignant ovarian cancer detection, with potentially superior performance compared to sonographers. Despite high heterogeneity across studies and the observed publication bias, these results indicate the potential for AI integration into clinical practice. Further studies with external, multicenter prospective head-to-head design are still needed.

## Introduction

Ovarian cancer is the deadliest form of cancer affecting the female reproductive system. Its early clinical symptoms are often subtle, leading to diagnosis at advanced stages and resulting in poor prognosis (1, 2). It is the leading cause of mortality among gynecological cancers, surpassing cervical and endometrial cancers in terms of lethality, with a 10-year survival rate of only 35% across all stages (3). The insidious nature of ovarian cancer presents a significant diagnostic challenge, as subtle or non-specific symptoms often result in delayed clinical presentations. Early and accurate diagnosis is pivotal to reducing mortality, improving treatment outcomes, and minimizing unnecessary surgical procedures for patients presenting with ovarian masses.

Conventional diagnostic modalities for ovarian cancer include imaging tools such as computed tomography (CT), magnetic resonance imaging (MRI), serum biomarkers like CA125 and HE4, pathological biopsy, and ultrasound. CT and MRI are common non-invasive methods, but their ability to fully analyze tumor microenvironments is limited because human vision has natural limitations (3, 4). Moreover, variability in radiological interpretation often depends on the expertise of the operator, which adding a layer of subjectivity to diagnostic accuracy. Pathological biopsy, though definitive, is invasive and not always suitable for all patients (5, 6). Serum biomarkers, despite being widely used, often lack specificity, as elevated levels can occur in conditions unrelated to ovarian malignancies (7). Ultrasound, particularly transvaginal ultrasound, has emerged as one of the most accessible and cost-effective tools for ovarian tumor evaluation (5). However, conventional ultrasound diagnostics primarily rely on morphological imaging and visual assessment, which are significantly influenced by operator-dependent variability. Quantitative data embedded within ultrasound images, which reportedly carry predictive advantages over traditional imaging metrics, often remain underexplored in clinical settings (1, 8). Thus, while these diagnostic methods offer valuable insights, their limitations underscore the urgent need for innovative solutions to improve diagnostic precision and reliability.

Artificial intelligence (AI) has demonstrated remarkable potential in improving diagnostic performance, especially in the analysis of medical imaging such as ultrasound. By leveraging machine learning and deep learning technologies, AI can extract complex patterns from imaging data and provide quantitative assessments of radiographic features that are often imperceptible to the human eye (4, 9). Recent studies have demonstrated that AI-assisted diagnostic performance in ovarian cancer can achieve sensitivity and specificity rates as high as 81% and 92%, which outperforming traditional imaging-based diagnostics (1). AI-based models can enhance the interpretation of ultrasound images by systematically quantifying tumor characteristics such as lesion size, echotexture, and morphological irregularities (8). However, despite their promising potential, AI based on ultrasound applications face significant controversies. First, the generalizability of AI models needs further evaluation, particularly regarding their diagnostic performance in external validation sets (6). Second, the relative diagnostic performance of AI compared to sonographers also requires assessment (10). These challenges underscore the necessity for systematic evaluations to clarify the performance variability of AI-enabled ultrasound diagnostics for ovarian cancer.

Therefore, this meta-analysis aims to systematically assess the diagnostic performance of AI-based ultrasound models for the initial diagnosis of ovarian cancer, and compare its performance to that of sonographers.

## Methods

The meta-analysis was conducted in strict accordance with the Preferred Reporting Items for Systematic Reviews and Meta-Analyses of Diagnostic Test Accuracy (PRISMA-DTA) guidelines (11).

### Search strategy

A systematic search was conducted across PubMed, Embase, Cochrane Library, and Web of Science up to February 2025. The search strategy integrated three conceptual domains: AI (e.g., “deep learning,” “machine learning”, “ artificial intelligence”), ovarian cancer (e.g., “Ovarian Neoplasms,” “Cancer of the Ovary”), and ultrasound imaging (e.g., “ultrasonography,” “Echography”). Free terms and MeSH headings were combined for comprehensive coverage (**Supplementary Table 1**). In addition, reference lists of included studies were manually screened. In order to minimize selection bias, the search was updated in March 2025 to include newly published studies.

### Inclusion and exclusion criteria

Inclusion criteria were established following the PICOS framework. Population (P): Patients suspected of ovarian malignancy or borderline ovarian tumors, which were classified as the malignant (positive) group in our study. Intervention (I): Application of AI algorithms to predict malignancy using ovarian ultrasound images, including transvaginal, transabdominal and transrectal approaches. Comparison (C): Pathological outcomes was reference standard. Outcomes (O): Studies reporting diagnostic performance metrics, including sensitivity, specificity, and AUC were included. Study design (S): Retrospective or prospective studies published in peer-reviewed journals.

Exclusion criteria encompassed: (1) studies lacking sufficient data to calculate true positive (TP), false positive (FP), false negative (FN), and true negative (TN) values; (2) non-relevant publication types (e.g., case reports, conference abstracts, reviews, meta-analyses, or commentaries); (3) non-English literature; (4) studies utilizing non-ultrasound-based AI methodologies (e.g., CT or MRI); (5) studies employing non-pathological reference standards.

### Quality assessment

The methodological quality of the included studies was assessed using a modified version of the Quality Assessment of Diagnostic Accuracy Studies-2 (QUADAS-2) tool (12). To enhance relevance, we integrated specific domain criteria from the Prediction model Risk of Bias Assessment Tool (PROBAST) to replace less applicable components of the original QUADAS-2 framework (13). The revised tool evaluated four domains: participant selection, index test (AI algorithm), reference standard, and analysis. Within each domain, risk of bias and concerns regarding applicability were systematically assessed. Two independent reviewers (X.T. and S.Z.) conducted the assessments using the modified QUADAS-2 tool. Disagreements were resolved through iterative discussions to ensure consensus.

### Data extraction

Two independent reviewers (R.L. and J.L.) conducted preliminary screening of titles and abstracts from the remaining literature to assess potential eligibility. Discrepancies in evaluations were resolved through adjudication by a third reviewer (H.Z.). Data extraction items included: author, publication year, country, study design, type of ultrasound, reference standard, analysis, patients/lesions/images per set, number of malignant ovarian cancer patients/lesions/images, AI method, AI model, optimal AI algorithms, data splitting method, the diagnostic matrix for internal and external validation sets, sonographers, scanner modality (system), evaluation time, and frequency (MHz).

For studies included in the systematic review but lacking meta-analyzable data, the research team contacted corresponding authors via email to obtain missing information. As most studies did not report complete diagnostic contingency tables, two methods were employed to construct the contingency table: (1) back-calculating total cases, along with sensitivity, specificity, and the number of malignant ovarian cancer patients based on the reference standard; (2) determining optimal sensitivity and specificity parameters through receiver operating characteristic (ROC) curve analysis using Youden’s index.

### Outcome measures

The primary outcome measures included sensitivity, specificity, and AUC from internal validation sets, external validation sets, and sonographer. Sensitivity (also termed recall or true positive rate) quantified the probability of the AI model correctly identifying malignant cases (including both ovarian cancer and borderline ovarian tumors), calculated as TP/(TP+FN). Specificity (true negative rate) represented the model’s ability to accurately identify non-malignant cases, computed as TN/(TN+FP). AUC, derived from the ROC curve, served as a composite metric for diagnostic discriminative performance. For studies reporting multiple contingency tables across datasets (e.g., two external validation cohorts), all independent datasets were extracted. When multiple AI models or algorithms were presented, in order to avoid patient overlap, only the optimal-performing model (highest AUC) from internal/external validation sets or sonographer comparisons was included.

### Statistical analysis

Statistical analyses were conducted using a bivariate random-effects model to pool sensitivity and specificity estimates for AI performance across internal validation, external validation, and sonographer datasets (14). Forest plots visually summarized pooled sensitivities and specificities, while SROC curves illustrated combined estimates with 95% confidence intervals (CIs) and prediction intervals. Heterogeneity between studies was quantified using the I^2^ statistic, with a threshold of 50% indicating possible significant heterogeneity (15). For internal validation datasets (>10 studies with I^2^ > 50%), subgroup analysis and meta-regression were conducted, evaluating covariates such as study design, analysis type, AI method, ultrasound type, AI model, and optimal AI algorithms. Clinical utility was assessed using Fagan’s nomogram, while publication bias was evaluated via Deeks’ funnel plot asymmetry test (16). The 95% CIs of the AUCs of AI and sonographer were compared. Nonoverlapping 95% CIs between two subgroups indicated a statistically significant difference. All analyses were conducted in Stata 15.1 (Midas package), with statistical significance defined as *P* < 0.05. Study quality and risk of bias were conducted using RevMan 5.4 (Cochrane Collaboration).

## Results

### Study selection

**Figure 1:**
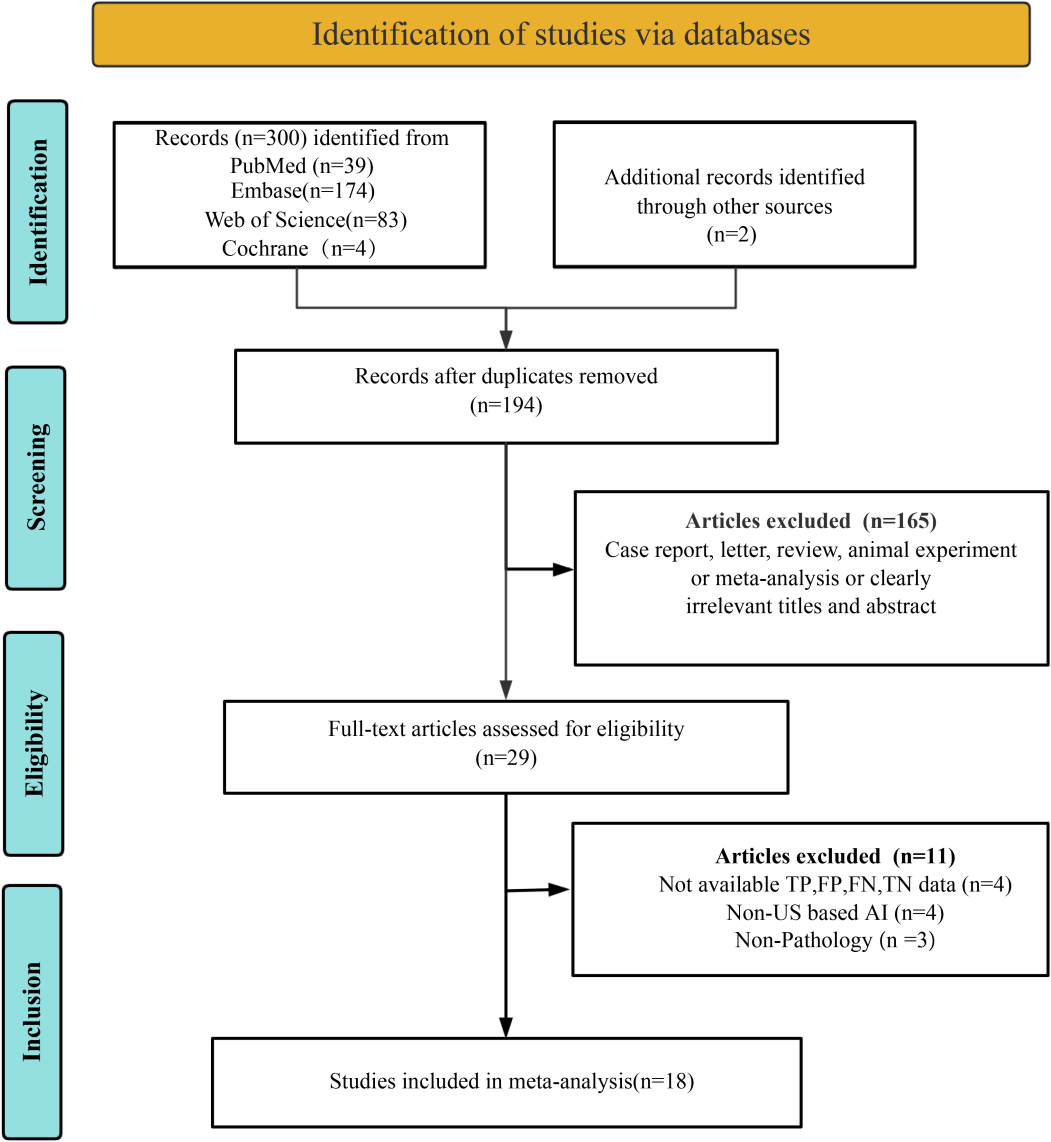
Preferred Reporting Items for Systematic Reviews and Meta-Analyses (PRISMA) flow diagram depicting the study selection process.

A systematic search of four databases identified 302 studies, with 108 duplicates removed, yielding 194 unique records for initial screening. A total of 165 articles were excluded during title/abstract screening due to non-relevant publication types (e.g., case reports, conference abstracts, reviews, meta-analyses, or commentaries) or clearly irrelevant titles/abstracts. Full-text eligibility assessment of the remaining 29 studies excluded 11 additional articles: four lacked extractable diagnostic performance metrics (TP, FP, FN, TN), four utilized non-ultrasound-based AI models, and three employed non-pathological reference standards. Eighteen studies met all criteria for final inclusion (9, 17–32). The selection process adhered to PRISMA guidelines, as detailed in **Figure 1**.

### Study description and quality assessment

A total of 18 eligible studies were identified, including 17 studies (7, 9, 17–21, 23–32) in the internal validation set with 22,697 total patients/images/lesions(range: 1-7,995), and three studies (9, 17, 22) in external validation involving 2,297 patients(range:2-662). The studies were published between 1999 and 2024. Among them, 13 studies included in the meta-analysis were retrospective (7, 9, 17–19, 21, 23, 26–28, 30–32), while five were prospective (20, 22, 24, 25, 29). Transvaginal ultrasound alone was used in nine studies (18–20, 22, 24, 25, 28, 30, 32), combined transvaginal and transabdominal ultrasound in four (7, 17, 21, 26), transabdominal ultrasound alone in one (31), transvaginal combined with transrectal ultrasound in one (9), and unspecified methods in three studies (23, 27, 29). Radiomic & Clinical AI models were employed in eight studies (7, 20, 22, 24–26, 29, 30), while radiomic models alone were used in ten studies (9, 17–19, 21, 23, 27, 28, 31, 32). Deep learning methods were utilized in eight studies (7, 9, 17, 19, 21, 23, 27, 31), with the remaining ten employing machine learning (18, 20, 22, 24–26, 28–30, 32). Patient-based analysis was conducted in nine studies (7, 9, 17, 19–22, 25, 33), lesion-based in four (18, 24, 29, 30), and image-based in five (23, 27, 28, 31, 32). Study characteristics along with patient demographics and technical details are summarized in **Tables 1**, **2**, and **Supplementary Table 2**.

**Table 1:** Study and patient characteristics of the included studies.

Author	Year	Country	Study design	Type of ultrasound	Type of histopathology	Reference standard	Analysis	Patients/lesions/images per set			No. of malignant ovarian cancer patients/lesions/images
								Training	Internal validation	External validation	
Li et al. (17)	2022	China	Retro	Transvaginal& Transabdominal	NA	Pathology	PB	1099	460	462	Training:217 Internal validation:71 External validation:84
Alcázar et al. (18)	2001	Spain	Retro	Transvaginal	NA	Pathology	LB	268	135	NA	Training:65 Internal validation:32
Szpurek et al. (19)	2005	Poland	Retro	Transvaginal	NA	Pathology	PB	500	66	NA	Training:185 Internal validation:22
Timmerman et al. (20)	1999	Belgium	Pro	Transvaginal	NA	Pathology	PB	116	57	NA	Training:33 Internal validation:16
Chen et al. (21)	2022	China	Retro	Transvaginal&Transabdominal	NA	Pathology	PB	296	85	NA	Training:83 Internal validation:24
Holsbeke et al. (22)	2007	multiple countries	Pro	Transvaginal	NA	Pathology	PB	NA	NA	809	External validation:242
Deeparani et al. (23)	2023	Sweden	Retro	NA	Epithelial ovarian cancer, non-epithelial ovarian cancer	Pathology	IB	23965	15977	NA	Training:11680 Internal validation:7995
Vaes et al.	2010	Belgium	Pro	Transvaginal	NA	Pathology	LB	NA	151	NA	Internal validation:83
Wang et al. (7)	2024	China	Retro	Transvaginal& Transabdominal	NA	Pathology	PB	675	210	NA	Training:228 Internal validation:70
Holsbeke et al. (25)	2009	multiple countries	Pro	Transvaginal	NA	Pathology	PB	NA	124	NA	Internal validation:26
Moro et al. (26)	2024	Italy	Retro	Transvaginal& Transabdominal	High grade serous	Pathology	PB	228	98	NA	Training:170 Internal validation:73
Jung et al. (27)	2022	Korea	Retro	NA	NA	Pathology	IB	1613	1613	NA	Training:539 Internal validation:539
Stefan et al. (28)	2021	Romania	Retro	Transvaginal	NA	Pathology	IB	NA	123	NA	Internal validation:35
Amidi et al. (29)	2019	America	Pro	NA	High grade serous carcinoma, Endometrioid carcinoma	Pathology	LB	NA	13	NA	Internal validation:7
Lin et al.	2024	America	Retro	Transvaginal	NA	Pathology	LB	NA	93	NA	Internal validation:21
Wang et al. (31)	2021	China	Retro	Transabdominal	Serous ovarian carcinoma	Pathology	IB	279	279	NA	Training:171 Internal validation:171
Acharya et al.	2012	NA	Retro	Transvaginal	NA	Pathology	IB	2600	2600	NA	Training:1300 Internal validation:1300
Gao et al. (9)	2022	China	Retro	Transvaginal & Transrectal	Serous, Mucinous, Endometrioid, Clear cell	Pathology	PB	105532	868	1224	Training:3755 Internal validation:266 External validation:233

tfn: Retro retrospective; Pro prospective; PB patient-based; IB image-based; LB lesion-based; NA not available.

**Table 2:** Technical aspects of included studies.

Author	Year	AI method	AI model	Optimal AI algorithms ^a^	Data splitting method	Internal validation sets				External validation sets				Sonographers			
						TP	FP	FN	TN	TP	FP	FN	TN	TP	FP	FN	TN
Li et al. (17) (set 1)	2022	Deep learning	Radiomic	CNN	Time-Series Split	62	14	9	120	46	9	5	42	41	9	10	42
Li et al. (17) (set 2)	2022	Deep learning	Radiomic	CNN	Time-Series Split	NA	NA	NA	NA	34	22	2	104	20	10	13	116
Alcázar et al. (18)	2001	Machine learning	Radiomic	LR	Random split	31	6	1	97	NA	NA	NA	NA	NA	NA	NA	NA
Szpurek et al. (19)	2005	Deep learning	Radiomic	MLP	Random split	18	3	4	41	NA	NA	NA	NA	NA	NA	NA	NA
Timmerman et al. (20)	1999	Machine learning	Radiomic &Clinical Model	LR	Random split	15	2	1	39	NA	NA	NA	NA	NA	NA	NA	NA
Chen et al. (21)	2022	Deep learning	Radiomic	CNN	Stratified sampling	22	9	2	52	NA	NA	NA	NA	23	8	1	53
Holsbeke et al. (22)	2007	Machine learning	Radiomic &Clinical Model	RVM	NA	NA	NA	NA	NA	218	146	24	421	NA	NA	NA	NA
Deeparani et al. (23)	2023	Deep learning	Radiomic	CNN	Random split	7995	3	2	7977	NA	NA	NA	NA	NA	NA	NA	NA
Vaes et al.	2010	Machine learning	Radiomic &Clinical	LR	Random split	69	1	14	67	NA	NA	NA	NA	NA	NA	NA	NA
Wang et al. (7)	2024	Deep learning	Radiomic &Clinical	CNN	Random split	66	7	4	133	NA	NA	NA	NA	NA	NA	NA	NA
Holsbeke et al. (25)	2009	Machine learning	Radiomic &Clinical	LR	Random split	25	25	1	73	NA	NA	NA	NA	NA	NA	NA	NA
Moro et al. (26)	2024	Machine learning	Radiomic &Clinical	LR	Random split	72	9	1	16	NA	NA	NA	NA	72	7	1	18
Jung et al. (27)	2022	Deep learning	Radiomic	CNN	5-fold cross validation	458	101	81	973	NA	NA	NA	NA	NA	NA	NA	NA
Stefan et al. (28)	2021	Machine learning	Radiomic	Multiple Regression	NA	32	6	3	82	NA	NA	NA	NA	NA	NA	NA	NA
Amidi et al. (29)	2019	Machine learning	Radiomic &Clinical	SVM	Random cross validation	5	1	2	5	NA	NA	NA	NA	NA	NA	NA	NA
Lin et al.	2024	Machine learning	Radiomic &Clinical	KNN	Random split	20	4	1	68	NA	NA	NA	NA	NA	NA	NA	NA
Wang et al. (31)	2021	Deep learning	Radiomic	DCNN	3-fold cross validation	156	10	15	98	NA	NA	NA	NA	128	19	43	89
Acharya et al.	2012	Machine learning	Radiomic	PNN	10-fold cross validation	1290	4	10	1296	NA	NA	NA	NA	NA	NA	NA	NA
Gao et al. (9) (set 1)	2022	Deep learning	Radiomic	DCNN	NA	210	41	56	561	27	4	40	264	44	46	36	254
Gao et al. (9) (set 2)	2022	Deep learning	Radiomic	DCNN	NA	NA	NA	NA	NA	96	61	70	662	NA	NA	NA	NA

TP, true positive; TN, true negative; FP, false positive; FN, false positive; NA, not available; ^a^ Optimal means the artificial intelligence algorithm with the highest AUC value; DCNN, Deep Convolutional Neural Networks; CNN, Convolutional Neural Networks; PNN, Probability Neural Network; KNN, k-Nearest Neighbors; MLP, Multilayer Perceptron; RVM, Relevance Vector Machine; SVM, Support Vector Machine; LR, Logistic Regression.

**Figure 2:**
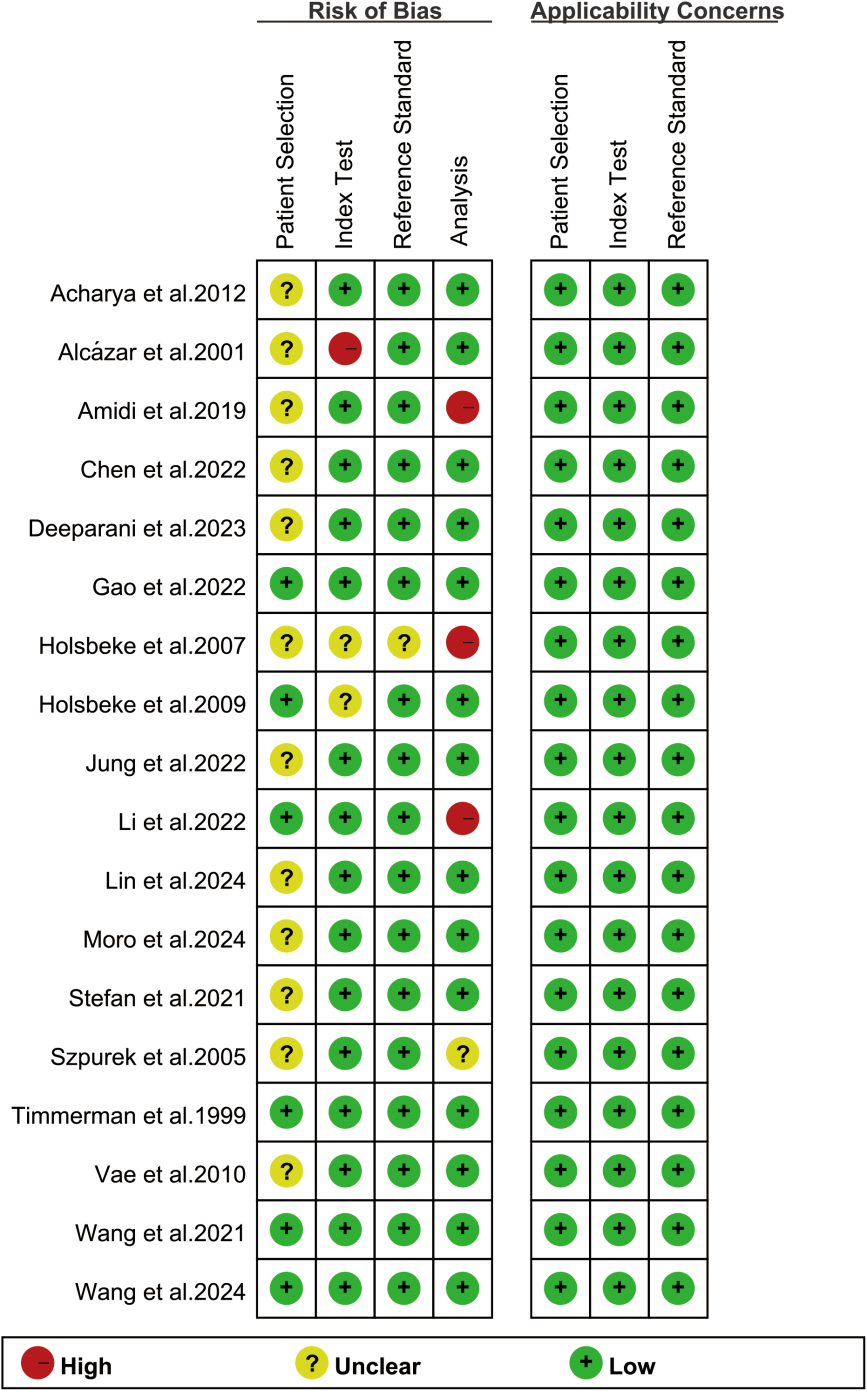
Risk of bias and applicability concerns of the included studies using the revised Quality Assessment of Diagnostic Performance Studies-2 (QUADAS-2) tool.

The risk of bias assessment using the revised QUADAS-2 tool is shown in **Figure 2**. Regarding the index test, one study was rated as “high risk” because it only reported the model name without critical training procedures details (18). In the analysis domain, three studies were deemed “high risk” due to the exclusion of participants from specific subgroups or partial cohorts (17, 22, 29). The methodological quality of included studies was deemed acceptable based on overall quality assessment.

### Diagnostic performance of internal validation sets for AI and sonographers in predicting ovarian malignancy: non-head-to-head comparison

**Figure 3:**
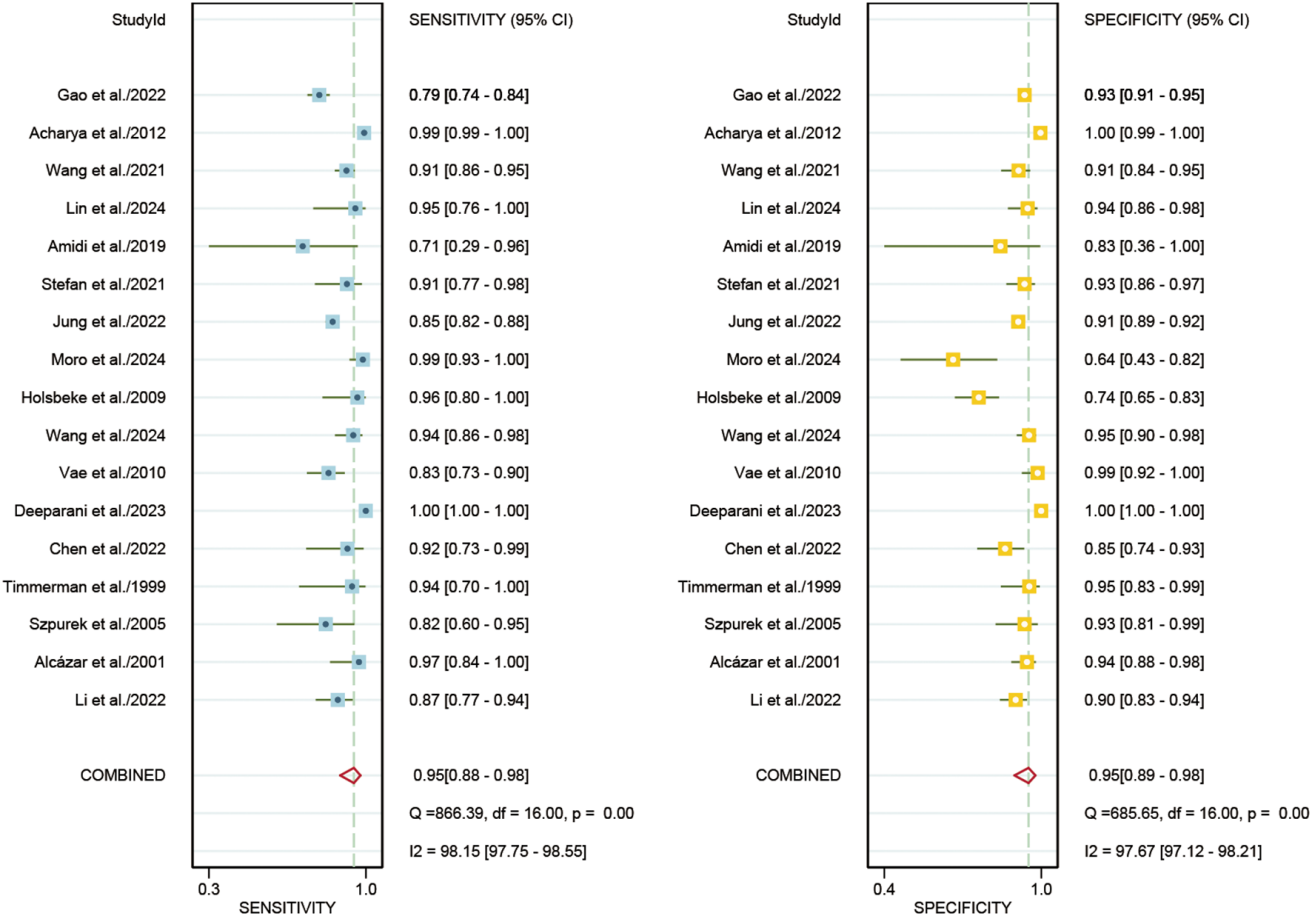
Forest plots displaying the sensitivity and specificity of the internal validation sets. Squares denoted the sensitivity and specificity in each study, while horizontal bars indicated the 95% confidence interval.

**Figure 4:**
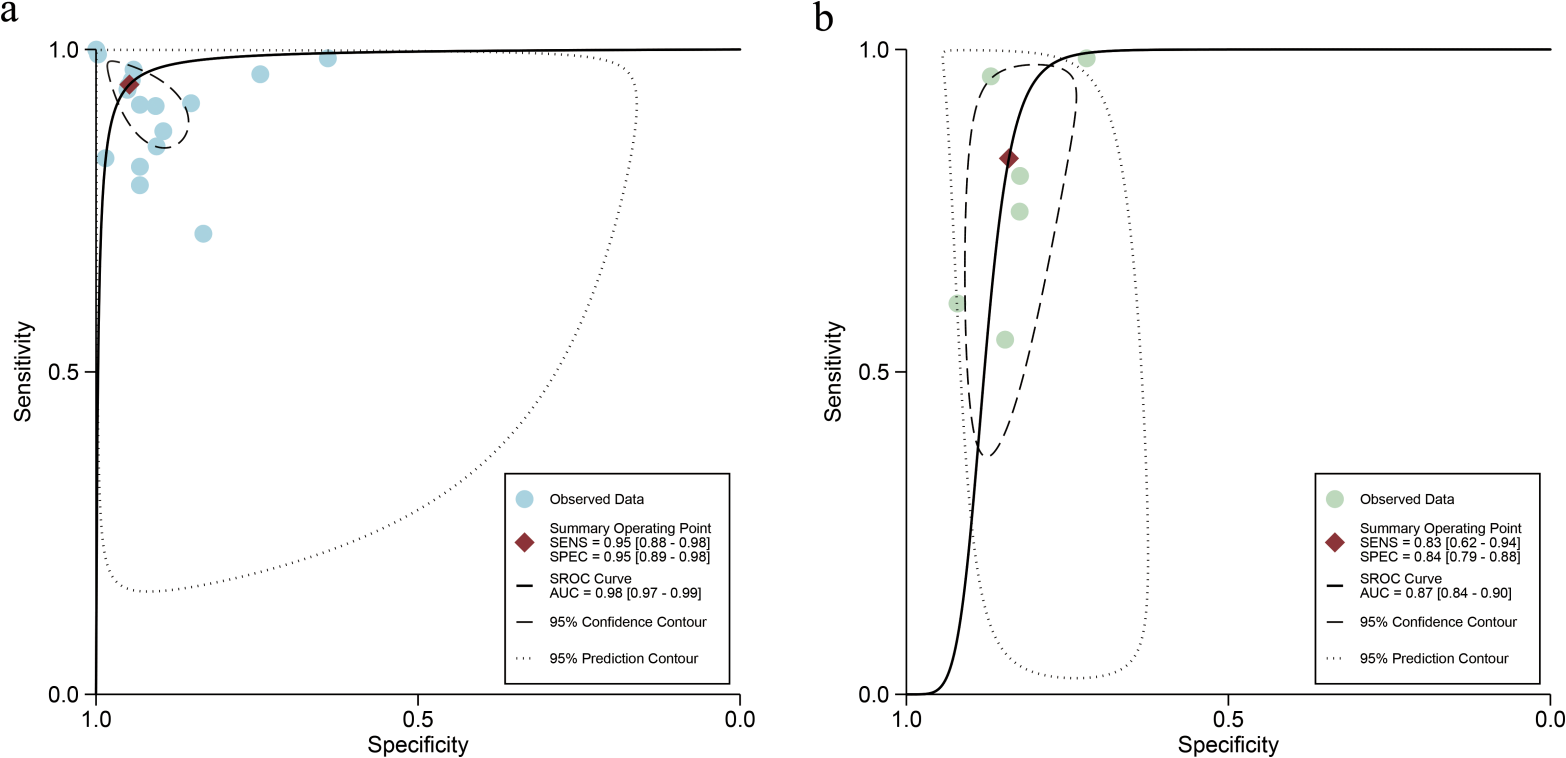
Summary receiver operating characteristic (SROC) curves of ultrasound-based artificial intelligence on the internal validation set **(a)** and sonographers **(b)** for diagnosing ovarian cancer.

**Figure 5:**
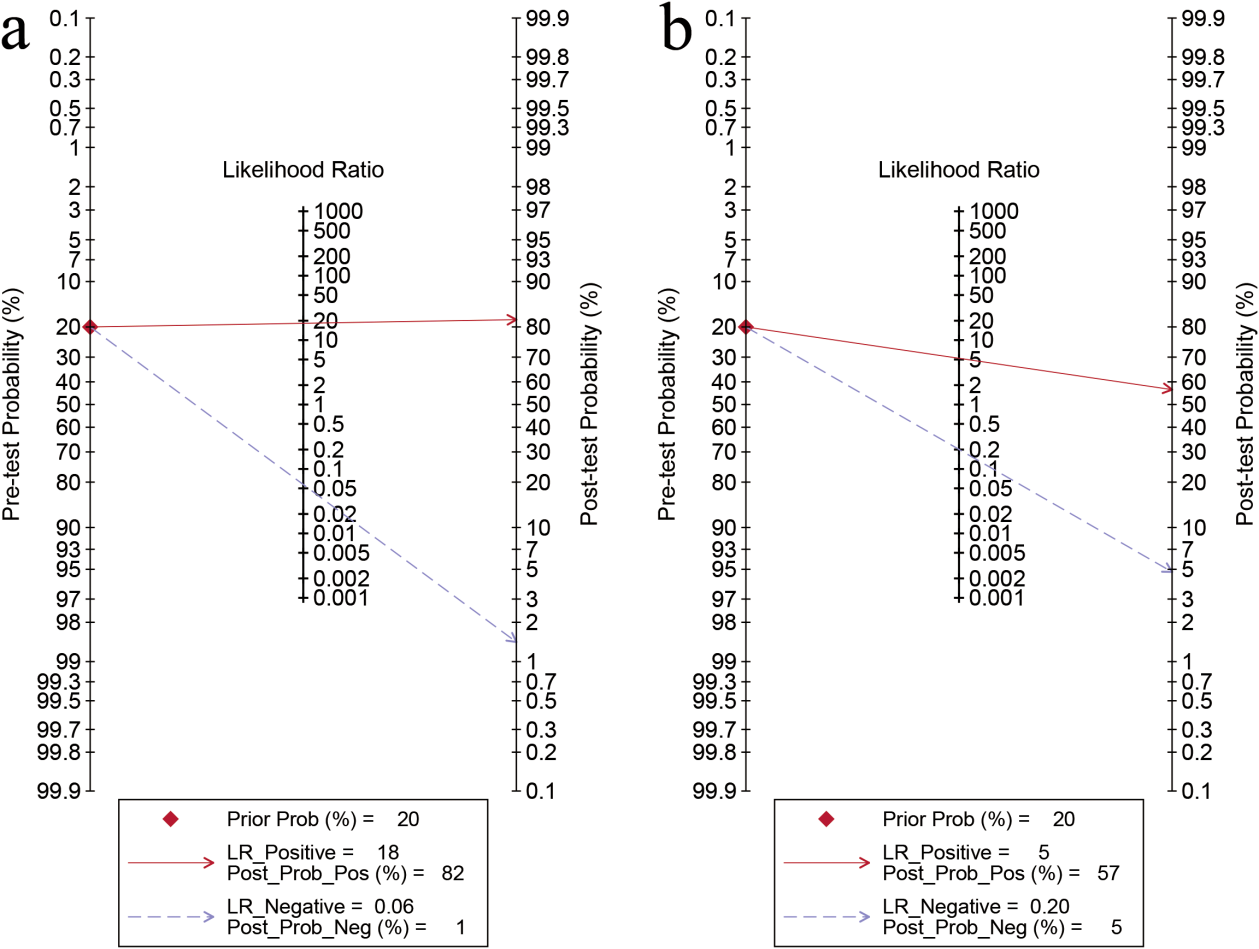
Fagan’s nomogram for artificial intelligence on the internal validation set **(a)** and sonographers **(b)** for diagnosing ovarian cancer.

For the internal validation sets, the sensitivity of AI based on ultrasound in detecting ovarian malignancy was 0.95 (95% CI: 0.88-0.98), and the specificity was 0.95 (95% CI: 0.89-0.98) (**Figure 3**). The AUC was 0.98 (95% CI: 0.97-0.99) (**Figure 4a**). Using a pretest probability of 20%, the Fagan nomogram showed a positive likelihood ratio of 82% and a negative likelihood ratio of 1% (**Figure 5a**).

**Figure 6:**
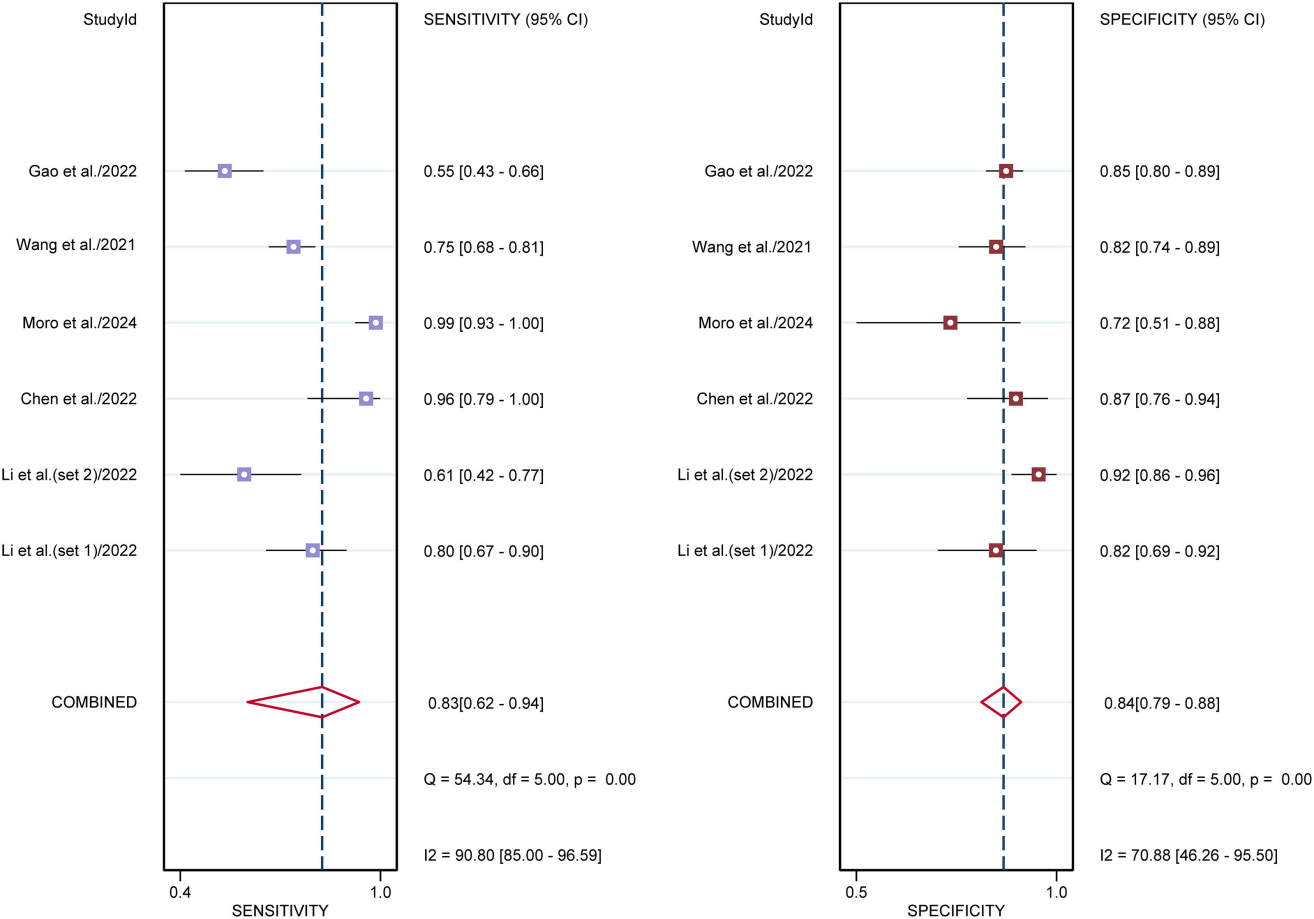
Forest plots displaying the sensitivity and specificity of the sonographers. Squares denoted the sensitivity and specificity in each study, while horizontal bars indicated the 95% confidence interval.

For sonographers, the sensitivity in detecting ovarian malignancy was 0.83 (95% CI: 0.62-0.94), and the specificity was 0.84 (95% CI: 0.79-0.88) (**Figure 6**). The AUC was 0.87 (95% CI: 0.84-0.90) (**Figure 4b**). Using a pretest probability of 20%, the Fagan nomogram showed a positive likelihood ratio of 57% and a negative likelihood ratio of 5% (**Figure 5b**). The AUC value of the AI based on ultrasound was significantly higher than that of the sonographers, with no overlapping 95% CI.

### Diagnostic performance of AI and sonographers in predicting ovarian malignancy: head-to-head comparison

Six studies provided data for head-to-head comparison. The sensitivity of AI based on ultrasound in detecting ovarian malignancy was 0.91 (95% CI: 0.74-0.97), and the specificity was 0.89 (95% CI: 0.76-0.95), and the AUC was 0.95 (95% CI: 0.93-0.97) (**Table 3**). For sonographers, the sensitivity in detecting ovarian malignancy was 0.83 (95% CI: 0.62-0.94), and the specificity was 0.84 (95% CI: 0.79-0.88). The AUC was 0.87 (95% CI: 0.84-0.90) (**Table 3**). The AUC value of the AI based on ultrasound was significantly higher than that of the sonographers, with no overlapping 95% CI.

**Table 3:** Sensitivity analysis of ultrasound-based artificial intelligence performance.

Sensitivity analysis	Datasets, n	Sensitivity (95%CI)	Specificity (95%CI)	AUC (95%CI)
Only patient-based analysis studies included	8	0.92(0.84-0.96)	0.88(0.82-0.93)	0.96(0.94-0.97)
Only image-based analysis studies included	5	0.98(0.85-1.00)	0.99(0.90-1.00)	1.00(0.99-1.00)
Excluding studies that included borderline ovarian tumors	2	0.99(0.98-0.99)	0.99(0.99-1.00)	NA
AI performance in head-to-head studies	6	0.91(0.74-0.97)	0.89(0.76-0.95)	0.95(0.93-0.97)
Sonographers performance in head-to-head studies	6	0.83(0.62-0.94)	0.84(0.79-0.88)	0.87(0.84-0.90)

AUC, area under the curve.

### Subgroup analysis and meta-regression for internal validation sets for AI in predicting ovarian malignancy

Subgroup analyses demonstrated consistently high sensitivity across all categories (range: 0.89-0.98), with no statistically significant differences observed between study designs (prospective vs. retrospective), AI methodologies (deep learning vs. machine learning), ultrasound approaches (transvaginal+ abdominal vs. transvaginal alone), AI model types (radiomic vs. radiomic+ clinical), or algorithm choices (CNN vs. logistic regression), or type of histopathology (serous vs. non-serous carcinomas) (all *P* > 0.05). Specificity values showed greater variability (range: 0.87-0.96), with a statistically significant difference noted between image-based (0.96, 95% CI: 0.93-1.00) and patient-based analyses (0.89, 95% CI: 0.79-1.00; *P* = 0.01). No other significant specificity differences were detected across subgroups including histological subtypes (all *P* > 0.05), though radiomic models (0.96) trended toward higher specificity than radiomic+ clinical models (0.90; *P* = 0.06).

For the internal validation sets, high heterogeneity was detected in both sensitivity (I² = 98.15%) and specificity (I² = 97.67%). Meta-regression analysis indicated that the heterogeneity was primarily attributed to the analysis method (Image-based vs. Patient-based, specificity *P* = 0.01) (**Table 4**).

**Table 4:** Subgroup analysis of artificial intelligence performance in internal validation sets for ovarian cancer.

Subgroup	Studies, n	Sensitivity(95%CI)	Meta-regression *P*-value	Specificity(95%CI)	Meta-regression *P*-value
Study design			0.34		0.51
Prospective	4	0.89(0.72-1.00)		0.88(0.83-0.93)	
Retrospective	13	0.96(0.92-0.99)		0.96(0.92-0.99)	
Analysis			0.09		0.01
Image-based	5	0.98(0.95-1.00)		0.96(0.93-1.00)	
Patient-based	8	0.92(0.84-1.00)		0.89(0.79-1.00)	
AI method			0.70		0.27
Deep learning	8	0.94(0.88-1.00)		0.96(0.91-1.00)	
Machine learning	9	0.95(0.90-1.00)		0.94(0.87-1.00)	
Type of Ultrasound			0.24		0.62
Transvaginal& Transabdominal	4	0.94(0.89-1.00)		0.87(0.74-1.00)	
Transvaginal	8	0.95(0.91-0.99)		0.96(0.92-0.99)	
AI model			0.39		0.06
Radiomic	10	0.95(0.91-1.00)		0.96(0.93-1.00)	
Radiomic & Clinical	7	0.93(0.85-1.00)		0.90(0.80-1.00)	
Optimal AI algorithms ^a^			0.69		0.32
CNN	7	0.95(0.89-1.00)		0.96(0.91-1.00)	
LR	5	0.96(0.88-1.00)		0.90(0.77-1.00)	
Type of histopathology					
Non-serous carcinoma	12	0.93(0.87-0.99)	0.67	0.94(0.89-0.99)	0.97
Serous carcinoma	5	0.97(0.92-1.00)		0.96(0.89-1.00)	

CNN, Convolutional Neural Networks; AI, Artificial Intelligence; ^a^ Optimal means the artificial intelligence algorithm with the highest AUC value; LR, Logistic Regression.

### Diagnostic performance of external validation sets for AI in predicting ovarian malignancy

For the external validation sets, the sensitivity of AI in detecting ovarian malignancy was 0.78 (95% CI:0.56-0.91), and the specificity was 0.88 (95% CI: 0.76-0.95) (**Supplementary Figure 1**). The AUC was 0.91 (95% CI: 0.88-0.93) (**Supplementary Figure 2**). Using a pretest probability of 20%, the Fagan nomogram showed a positive likelihood ratio of 63% and a negative likelihood ratio of 6% (**Supplementary Figure 3**).

### Publication bias

**Figure 7:**
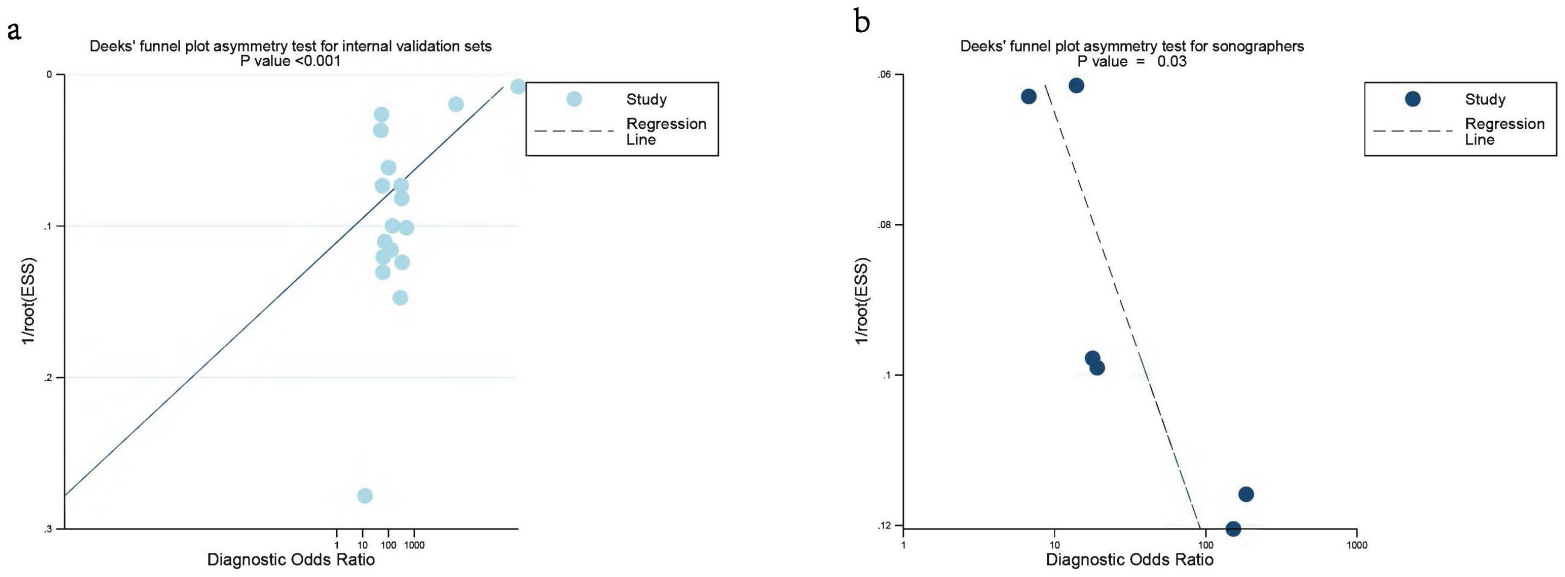
Deek’s funnel plot was used to evaluate internal validation set **(a)** and sonographers **(b)** the publication bias of ultrasound-based artificial intelligence for diagnosing ovarian cancer. *P* < 0.05 was considered significant.

Deeks’ funnel plot asymmetry test indicated significant publication bias in the internal validation sets for AI and sonographers (*P* < 0.001, *P* = 0.03) (**Figures 7a, b**). However, no significant publication bias was found in the external validation sets (*P* = 0.13) (**Supplementary Figure 4**).

## Discussion

Ultrasound-based AI demonstrated excellent diagnostic performance in the initial diagnosis of ovarian cancer, achieving AUC values of 0.98 and 0.91 in the internal and external validation sets, respectively. Additionally, our results revealed that the AUC value for sonographers was 0.87, with no overlap in the 95%CI compared to AI, indicating that the AI model significantly outperformed traditional ultrasound diagnosis. The decline in diagnostic performance in the external validation sets may be attributed to sample heterogeneity and differences in operational standards across hospitals, which could affect the model’s generalizability. In contrast, the internal validation sets was conducted in a relatively uniform environment, ensuring the model’s high performance (1, 2). The advantages of ultrasound-based AI algorithms in complex pattern recognition and data processing enable them to better identify subtle lesion features, thereby providing higher sensitivity and specificity. However, sonographers may be influenced by factors such as experience, fatigue, and subjective judgment when interpreting images, leading to lower diagnostic performance (3, 9). Therefore, AI technology in the early screening of ovarian cancer may effectively improve diagnostic accuracy and enhance patient prognosis.

Interestingly, according to the results of our subgroup analysis, the sensitivity and specificity of deep learning were 0.94 and 0.96, respectively, while those of machine learning were 0.95 and 0.94. The differences between the two were not statistically significant (both *P* > 0.05). This indicates that deep learning does not significantly outperform machine learning, which is consistent with Xu et al. previous findings (1). However, due to the high heterogeneity, these results should be interpreted with caution. More studies are needed in the future to confirm whether deep learning algorithms offer any additional value over machine learning algorithms in the accurate diagnosis of ovarian cancer.

We found that regarding different AI models, the sensitivity and specificity of radiomic models did not show statistically significant differences compared to the combined approach of radiomic & clinical models (both *P* > 0.05). This may be attributed to the inherent complexities of integrating clinical data with imaging features (34). Radiomic, which extracts quantitative features from medical images, may not fully account for the multifactorial nature of tumor behavior and clinical symptoms that influence diagnostic outcomes (35). Furthermore, the potential redundancy in the information provided by radiomic features and clinical data may not contribute to an improvement in diagnostic performance metrics (2). In the studies we included, most of the researches that combined radiomics with clinical features used machine learning algorithms, while studies that relied solely on radiomics mostly adopted deep learning algorithms. Therefore, the choice of training algorithm may also influence the current results. More research is needed in the future to determine the value of multimodal AI algorithms compared to studies using radiomics alone.

In 2022, Xu et al. (1) performed a meta-analysis evaluating the diagnostic performance of a multimodal imaging-based AI approach incorporating CT, MRI, and ultrasound for ovarian cancer. The AI-based ultrasound diagnosis demonstrated a sensitivity of 0.91, specificity of 0.87, and AUC of 0.95. Their research compared a multimodal imaging-based AI approach incorporating CT, MRI, and ultrasound with human radiologists and found that AI outperformed the radiologists (AUC: 0.93 vs. 0.85). In 2024, Mitchell et al. (3) published another meta-analysis on AI-based ultrasound diagnosis for ovarian cancer, reporting an AI sensitivity of 0.81, specificity of 0.92, and AUC of 0.87. However, only two of the included studies compared AI with sonographers, and the diagnostic performance of sonographers was not extracted in their study. In addition, their study only performed a pooled analysis of 14 articles and did not conduct in-depth subgroup analyses based on different ultrasound equipment types or various AI algorithms.

To the best of our knowledge, this the first meta-analysis to separately extract data from internal validation sets, external validation sets, and diagnostic performance data of sonographers. Compared to previous published studies, our results demonstrate higher diagnostic performance for AI models (AUC:0.98), which may be attributed to updates in new algorithms that enhance performance. Our findings also indicate that AI-based ultrasound exhibits a diagnostic performance advantage over sonographers in the initial diagnosis of ovarian cancer (AUC:0.98 vs. 0.87), which consistent with prior findings (1).

It’s worth to noted that the inclusion of studies in our meta-analysis demonstrated significant heterogeneity, potentially impacting the overall sensitivity and specificity of AI performance. We conducted a meta-regression analysis, revealing that one of the primary sources of heterogeneity was the difference between analysis (Image-based vs. Patient-based *P* = 0.01). Image-based studies can provide more detailed information, leading to higher diagnostic performance, which may result in overestimation of the accuracy of findings. This is largely due to their focus on specific anatomical structures and lesions, allowing for a nuanced analysis that can highlight minute details that might be overlooked in broader studies. In contrast, patient-based studies do not capture as much information, resulting in significant differences in design and methodology. However, the high heterogeneity may also be attributed to other factors: (1) For patient characteristics, the different stages of ovarian tumors and the age of patients may also influence the performance; (2) For intervention methods, the quality of ultrasound imaging itself, which depends on operator expertise and equipment, could influence AI diagnostic accuracy. These factors highlight the need for standardized reporting in future studies to facilitate more robust meta-analyses.

Our results demonstrate that AI models based on ultrasound have achieved diagnostic performance in both internal and external validation datasets, and they have the potential to become important support tools for sonographers. AI can serve as a powerful auxiliary tool, offering physicians a valuable second opinion, enhancing diagnostic reliability, and reducing the risk of missed or incorrect diagnoses. Additionally, AI has the potential to decrease unnecessary surgeries or procedures, thereby helping to lessen the clinical workload, improve diagnostic accuracy, and prevent adverse outcomes caused by missed or delayed diagnoses (32). Implementing ultrasound-based AI in primary healthcare systems could facilitate early detection and timely management of ovarian cancer. However, it is crucial to emphasize that these models should not be regarded as standalone diagnostic standards or decision-making tools but rather as supplementary aids for sonographers (9). Due to the significant differences in algorithm interpretability and the lack of standardized algorithm training for ovarian cancer diagnosis, we can only conclude that our results represent a short-term phenomenon, indicating that current AI algorithms already have the potential to outperform traditional sonographers. Notably, only five studies in our analysis compared AI diagnostic performance directly with that of sonographers, highlighting the need for further research in this area.

Some limitations of the current meta-analysis should be considered when interpreting the results. First, most of the included studies were retrospective in design, with only four employing a prospective approach, which may introduce potential biases. To assess the possible impact of different study designs on the outcomes, we conducted a meta-regression analysis, which revealed that the type of study did not significantly affect the results (both P > 0.05). Therefore, well-designed prospective studies are essential to validate the findings of this meta-analysis and ensure the reliability and generalizability of the results. Second, only the optimal AI algorithms were selected for analysis, which may lead to an overestimation of the results. This was primarily because we aimed to avoid potential patient overlap. However, this approach may have led to an overly optimistic assessment of AI model performance. Future studies should compare the performance of different models, including those with moderate or poor outcomes, to provide a more comprehensive and objective evaluation. Third, due to lack of information, the study did not stratify sonographers by their experience and skill level. Future studies should consider stratifying sonographers by experience levels to better evaluate the impact of AI assistance on diagnostic performance across different practitioner groups. Fourth, since most of the included studies classified borderline tumors as part of the malignant (positive) group, we followed their approach and classified both borderline and malignant tumors as a positive group. However, this may have contributed to an increased rate of positive diagnoses. In the future, there is a need to evaluate AI’s ability to distinguish between benign, borderline, and malignant ovarian tumors to aid early clinical intervention for malignant cases. Forth, the very high heterogeneity (It > 97%) observed in both sensitivity and specificity, partly driven by methodological factors such as the unit of analysis, significantly limits the strength of conclusions that can be drawn regarding their current clinical readiness. Therefore, any claims about their ability to enhance diagnostic reliability, reduce workload, or improve patient outcomes in clinical practice are currently premature and require validation through more standardized, large-scale studies. Fifth, the classification of borderline tumors represents a potential source of heterogeneity. Most included studies categorized borderline tumors as malignant for the purpose of binary classification. While this reflects a common clinical challenge in distinguishing these entities from invasive cancer, it may inflate the perceived sensitivity of AI models and complicate the clinical interpretation of results, as the management of borderline tumors differs from that of frank malignancy. To address this concern, we performed a sensitivity analysis excluding studies that explicitly included borderline tumors in the malignant group. Future research should aim to evaluate AI performance across a three-tiered stratification (benign, borderline, malignant) to better assess its clinical utility in differentiating this diagnostically challenging spectrum of disease.

## Conclusion

AI based on ultrasound diagnosis demonstrates excellent performance for malignant ovarian cancer detection, with potentially superior performance compared to sonographers. Despite high heterogeneity across studies and the observed publication bias, these results indicate the potential for AI integration into clinical practice. Further studies with external, multicenter prospective head-to-head design are still needed.

## Data Availability

The original contributions presented in the study are included in the article/**Supplementary Material**. Further inquiries can be directed to the corresponding author.
