# Supplementary material for: Artificial intelligence based on ultrasound for initial diagnosis of malignant ovarian cancer: a systematic review and meta-analysis
Source: Front Oncol. 2025 Dec 1;15:1626286. doi: 10.3389/fonc.2025.1626286 (PMC12702728; doi:10.3389/fonc.2025.1626286)

Supplementary Table 1:Search strategy in PubMed, Web of Science, Embase, and Cochrane Library.

| Pubmed | | Total |
| --- | --- | --- |
| 1 | ((Artificial Intelligence[MeSH Terms] OR ((((Computational Intelligence[Title/Abstract]) OR (Computer Reasoning[Title/Abstract])) OR (Computer Vision System*[Title/Abstract])) OR (Machine Intelligence[Title/Abstract]))) OR ((Machine Learning[MeSH Terms]) OR (Transfer Learning[Title/Abstract]))) OR ((Deep Learning[MeSH Terms]) OR (Hierarchical Learning[Title/Abstract])) | 224,348 |
| 2 | (Ultrasonography[MeSH Terms]) OR (((((((((Diagnostic Ultrasound*[Title/Abstract]) OR (Echography[Title/Abstract])) OR (Echotomography[Title/Abstract])) OR (Medical Sonography[Title/Abstract])) OR (Ultrasonic Diagnos*[Title/Abstract])) OR (Ultrasonic Imaging[Title/Abstract])) OR (Ultrasonic Tomography[Title/Abstract])) OR (Ultrasonographic Imaging*[Title/Abstract])) OR (Ultrasound Imaging[Title/Abstract])) | 515,044 |
| 3 | (Ovarian Neoplasms[MeSH Terms]) OR (((((Cancer of the Ovary[Title/Abstract]) OR (Ovarian Cancer*[Title/Abstract])) OR (Ovarian Neoplasm[Title/Abstract])) OR (Ovary Cancer*[Title/Abstract])) OR (Ovary Neoplasm*[Title/Abstract])) | 124,807 |
|  | #1 AND #2 AND #3 | 39 |

| WOS | | Total |
| --- | --- | --- |
| 1 | ((((TS=(Artificial Intelligence)) OR TS=(Computational Intelligence)) OR TS=(Computer Reasoning)) OR TS=(Computer Vision System*)) OR TS=(Machine Intelligence) | 287758 |
| 2 | (TS=(Deep Learning)) OR TS=(Hierarchical Learning) | 432774 |
| 3 | (TS=(Machine Learning)) OR TS=(Transfer Learning) | 597802 |
| 4 | #3 OR #2 OR #1 | 1093032 |
| 5 | (((((((((TS=(Ultrasonography)) OR TS=(Diagnostic Ultrasound*)) OR TS=(Echography)) OR TS=(Echotomography)) OR TS=(Medical Sonography)) OR TS=(Ultrasonic Diagnos*)) OR TS=(Ultrasonic Imaging)) OR TS=(Ultrasonic Tomography)) OR TS=(Ultrasonographic Imaging*)) OR TS=(Ultrasound Imaging) | 307290 |
| 6 | (((((TS=(Ovarian Neoplasms)) OR TS=(Cancer of the Ovary)) OR TS=(Ovarian Cancer*)) OR TS=(Ovarian Neoplasm)) OR TS=(Ovary Cancer*)) OR TS=(Ovary Neoplasm*) | 160766 |
| 7 | #4 AND #5 AND #6 | 83 |

| EMBASE | | Total |
| --- | --- | --- |
| 1 | ('ultrasound'/exp OR 'phonophoresis':ti,ab,kw OR 'sonication':ti,ab,kw OR 'sonification':ti,ab,kw OR 'ultra sound':ti,ab,kw OR 'ultrashell':ti,ab,kw OR 'ultrasonic':ti,ab,kw OR 'ultrasonic energy':ti,ab,kw OR 'ultrasonic irradiation':ti,ab,kw OR 'ultrasonic measurement':ti,ab,kw OR 'ultrasonic sound':ti,ab,kw OR 'ultrasonic wave*':ti,ab,kw OR 'ultrasonics':ti,ab,kw OR 'ultrasound radiation') OR ('echography'/exp OR 'diagnostic ultrasonic examination':ti,ab,kw OR 'diagnostic ultrasonic imaging':ti,ab,kw OR 'diagnostic ultrasonic method':ti,ab,kw OR 'diagnostic ultrasound':ti,ab,kw OR 'doptone':ti,ab,kw OR 'duplex echography':ti,ab,kw OR 'echogram':ti,ab,kw OR 'echographic evaluation':ti,ab,kw OR 'echoscopy':ti,ab,kw OR 'echosound':ti,ab,kw OR 'high resolution echography':ti,ab,kw OR 'sonogram':ti,ab,kw OR 'sonographic examination':ti,ab,kw OR 'sonographic screening':ti,ab,kw OR 'sonography':ti,ab,kw OR 'ultrasonic detection':ti,ab,kw OR 'ultrasonic diagnosis':ti,ab,kw OR 'ultrasonic echo':ti,ab,kw OR 'ultrasonic examination':ti,ab,kw OR 'ultrasonic scanning':ti,ab,kw OR 'ultrasonic scintillation':ti,ab,kw OR 'ultrasonogram':ti,ab,kw OR 'ultrasonographic examination':ti,ab,kw OR 'ultrasonographic screening':ti,ab,kw OR 'ultrasonography':ti,ab,kw OR 'ultrasound diagnosis':ti,ab,kw OR 'ultrasound scanning'):ti,ab,kw | 1429664 |
| 2 | 'ovary tumor'/exp OR 'neoplasm* of the ovary':ti,ab,kw OR 'neoplastic ovar*':ti,ab,kw OR 'ovarian neoplas*':ti,ab,kw OR 'ovarian tumo*':ti,ab,kw OR 'ovarium tumo*':ti,ab,kw OR 'ovary neoplasm':ti,ab,kw OR 'ovary tumo* treatment':ti,ab,kw OR 'ovary tumorigenesis':ti,ab,kw OR 'ovary tumour':ti,ab,kw OR 'tumo* of the ovary':ti,ab,kw | 207698 |
| 3 | ('machine learning'/exp OR 'learning machine*':ti,ab,kw) OR ('artificial intelligence'/exp OR 'machine intelligence':ti,ab,kw) OR ('deep learning'/exp OR 'deep machine learning':ti,ab,kw OR 'deep ml':ti,ab,kw OR 'hierarchical learning':ti,ab,kw) | 592691 |
|  | #1 AND #2 AND #3 | 174 |

| Cochrane | |  |
| --- | --- | --- |
| #1 | MeSH descriptor: [Artificial Intelligence] explode all trees | 3453 |
| #2 | MeSH descriptor: [Ultrasonography] explode all trees | 19740 |
| #3 | MeSH descriptor: [Ovarian Neoplasms] explode all trees | 3532 |
| #4 | MeSH descriptor: [Machine Learning] explode all trees | 1086 |
| #5 | MeSH descriptor: [Deep Learning] explode all trees | 358 |
| #6 | (Computational Intelligence):ti,ab,kw OR (Computer Reasoning):ti,ab,kw OR (Computer Vision System*):ti,ab,kw OR (Machine Intelligence):ti,ab,kw | 1071 |
| #7 | (Diagnostic Ultrasound* OR Echography OR Echotomography OR Medical Sonography OR Ultrasonic Diagnos* OR Ultrasonic Imaging OR Ultrasonic Tomography OR Ultrasonographic Imaging* OR Ultrasound Imaging):ti,ab,kw | 21374 |
| #8 | (‘Cancer of the Ovary OR Ovarian Cancer* OR Ovarian Neoplasm OR Ovary Cancer* OR Ovary Neoplasm*’):ti,ab,kw | 9932 |
| #9 | (#4 OR Transfer Learning):ti,ab,kw | 2787 |
| #10 | (#5 OR Hierarchical Learning):ti,ab,kw | 637 |
| #11 | (#1 OR #6) | 4349 |
| #12 | (#11 OR #10 OR #9) | 6268 |
| #13 | (#2 OR #7) | 33044 |
| #14 | (#3 OR #8) | 10178 |
| #15 | (#12 AND #13 AND #14) | 4 |

Supplementary Table 2: Technical aspects of ultrasound-based AI in included studies.

| Author | Year | Scanner Modality (System) | Menopausal state | Ultrasound Type | Frequency (MHz) |
| --- | --- | --- | --- | --- | --- |
| Li et al. | 2022 | 1.Mindray Resona8T  2.Mindray Resona7  3.GE VolusonE8  4. Philips EPIQ7  5. Samsung Medison WS80A  6. Hitachi  7.Siemens | Premenopausal | Transvaginal&Transabdominal | 2-12 |
| Alcázar et al. | 2001 | 1.Philips P-700 SE  2.Toshiba SSA-370 A Power Vision | Premenopausal& Postmenopausal | Transvaginal | 5-7.5 |
| Szpurek et al. | 2005 | 1.B-K Medical 3535  2.Aloka 2000/5500 | Premenopausal& Postmenopausal | Transvaginal | 5.0-6.5 |
| Timmerman et al. | 1999 | Acuson 128 XP/10 Acoustic | Postmenopausal | Transvaginal | 5.0 |
| Chen et al. | 2022 | 1.GE VolusonE10  2.Philips IU22  3.Philips A70 | Premenopausal& Postmenopausal | Transvaginal&Transabdominal | TVS:5.0-9.0 (GE)  4.0-8.0 (Philips IU22)  3.0-10.0 (Philips A70)  TUS:1.0-5.0 |
| Holsbeke et al. | 2007 | NA | Premenopausal& Postmenopausal | Transvaginal | NA |
| Deeparani et al. | 2023 | NA | Premenopausal& Postmenopausal | NA | NA |
| Vae et al. | 2010 | Voluson 730 Expert | Postmenopausal | Transvaginal | 5-9 |
| Wang et al. | 2024 | 1.GE VolusonE10/E9  2.Philips EPIQ7  3.Mindray Resona7 | Premenopausal& Postmenopausal | Transvaginal&Transabdominal | TVS:5.0-9.0 MHz  4.0-8.0 MHz  MHz  TUS:1.0-5.0 MHz |
| Holsbeke et al. | 2009 | NA | NA | Transvaginal | NA |
| Moro et al. | 2024 | 1.GE VolusonE10  2.Samsung Medison HERA W10/HERA I10 | NA | Transvaginal&Transabdominal | TVS:5.0-9.0  TUS:3.5-5.0 |
| Jung et al. | 2022 | Samsung Medison Accuvix XQ/WS80A Elite | NA | NA | NA |
| Stefan et al. | 2021 | Toshiba Medical Systems Aplio 300 | NA | Transvaginal | 4-10 |
| Amidi et al. | 2019 | Toshiba Medical Systems Alpinion EC-12R | NA | NA | 3.5-7 |
| Lin et al. | 2024 | Toshiba Medical Systems Alpinion E-CUBE 12 | NA | Transvaginal | NA |
| Wang et al. | 2021 | 1.Philips: PIQ5/EPIQ7/IU22  2.Samsung:RS80A  3.GE Healthcare: LOGIQ E9/LOGIQ S7 | NA | Transabdominal | NA |
| Acharya et al. | 2012 | NA | Premenopausal& Postmenopausal | Transvaginal | NA |
| Gao et al. | 2022 | 1.Toshiba Medical Systems Toshiba Aplio800  2.Siemens Acuson S3000  3.Philips EPIQ7 4.GE Voluson S8 | NA | Transvaginal & Transrectal | NA |

Supplementary Figure 1: Forest plot of diagnostic performance of ultrasound based-artificial intelligence external validation sets for initial diagnosis of malignant ovarian cancer. The sensitivity and specificity of each study were represented by squares, with horizontal bars indicating the 95% confidence intervals.


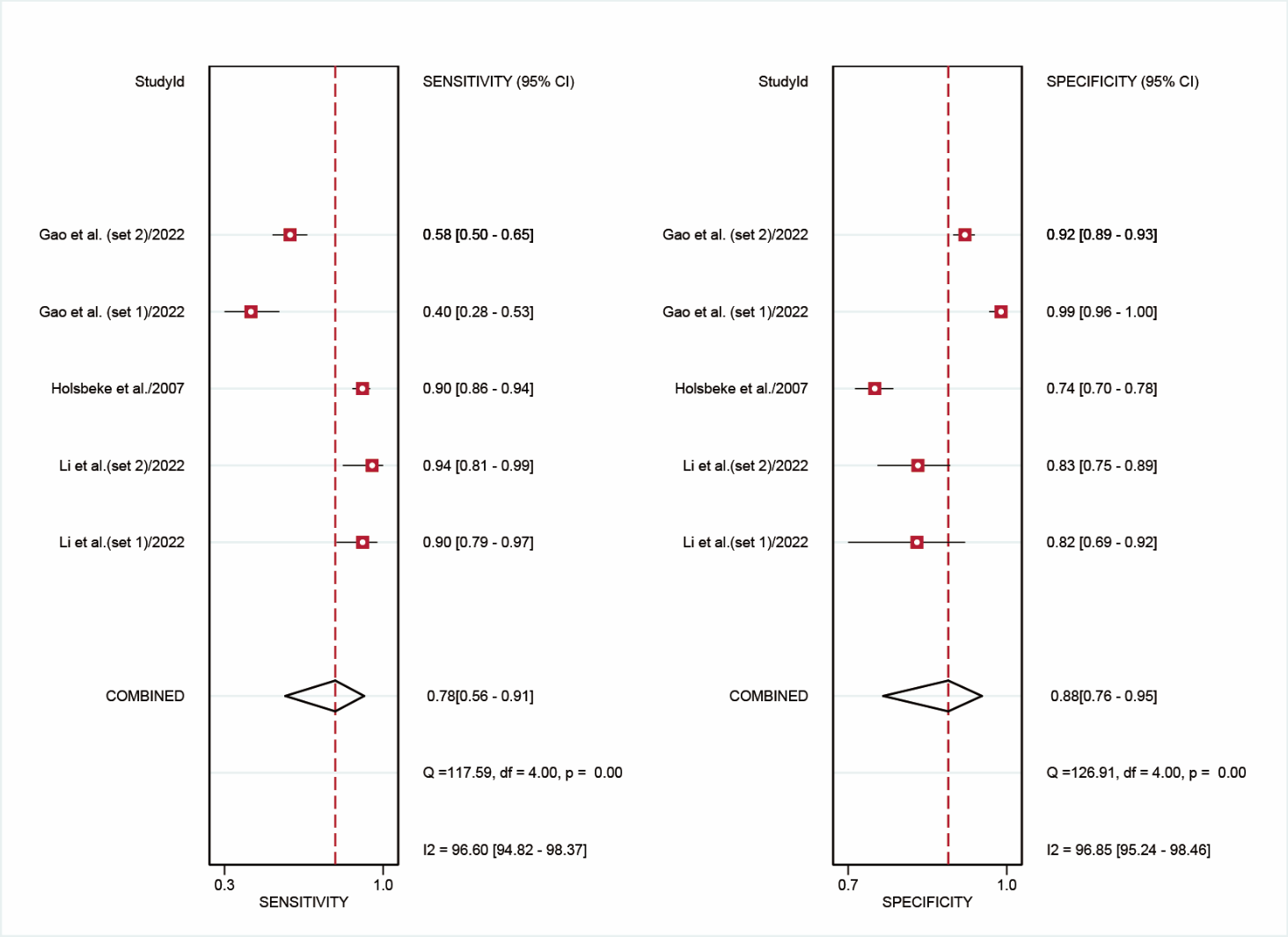


Supplementary Figure 2: Summary receiver operating characteristic (SROC) curves of diagnostic performance of ultrasound based-artificial intelligence external validation sets for initial diagnosis of malignant ovarian cancer.


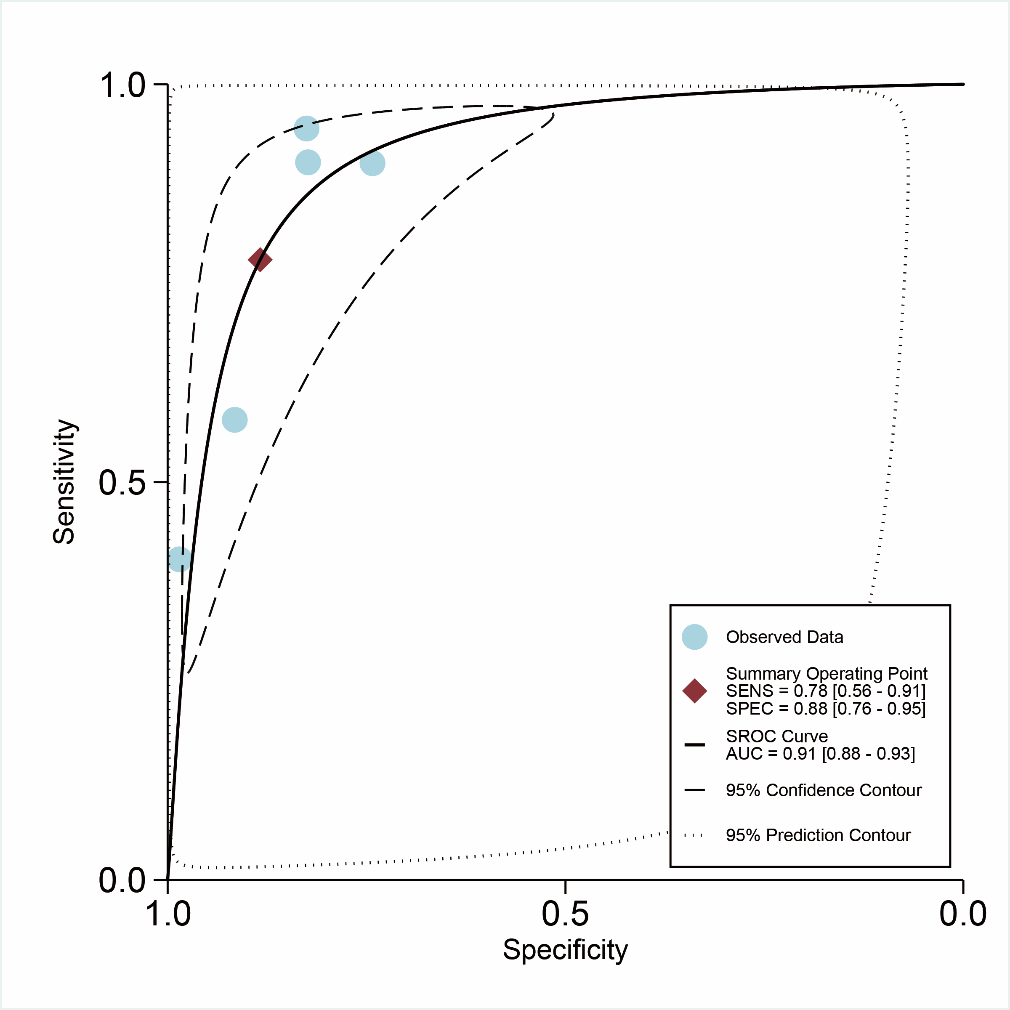


Supplementary Figure 3: Fagan plot of diagnostic performance of ultrasound based-artificial intelligence external validation sets for initial diagnosis of malignant ovarian cancer.


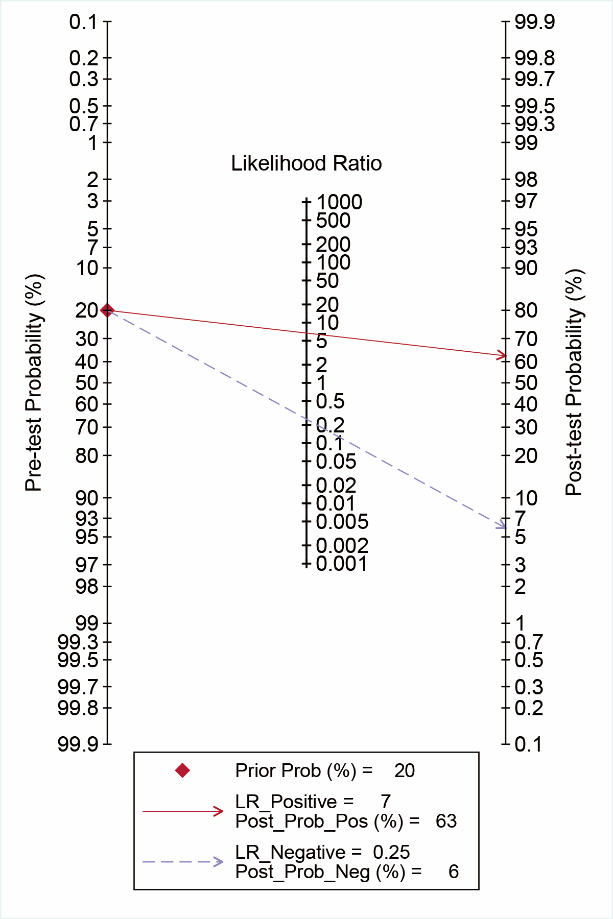


Supplementary Figure 4: Deek's funnel plot of diagnostic performance of ultrasound based-artificial intelligence external validation sets for initial diagnosis of malignant ovarian cancer. *P<*0.05 was considered significant.


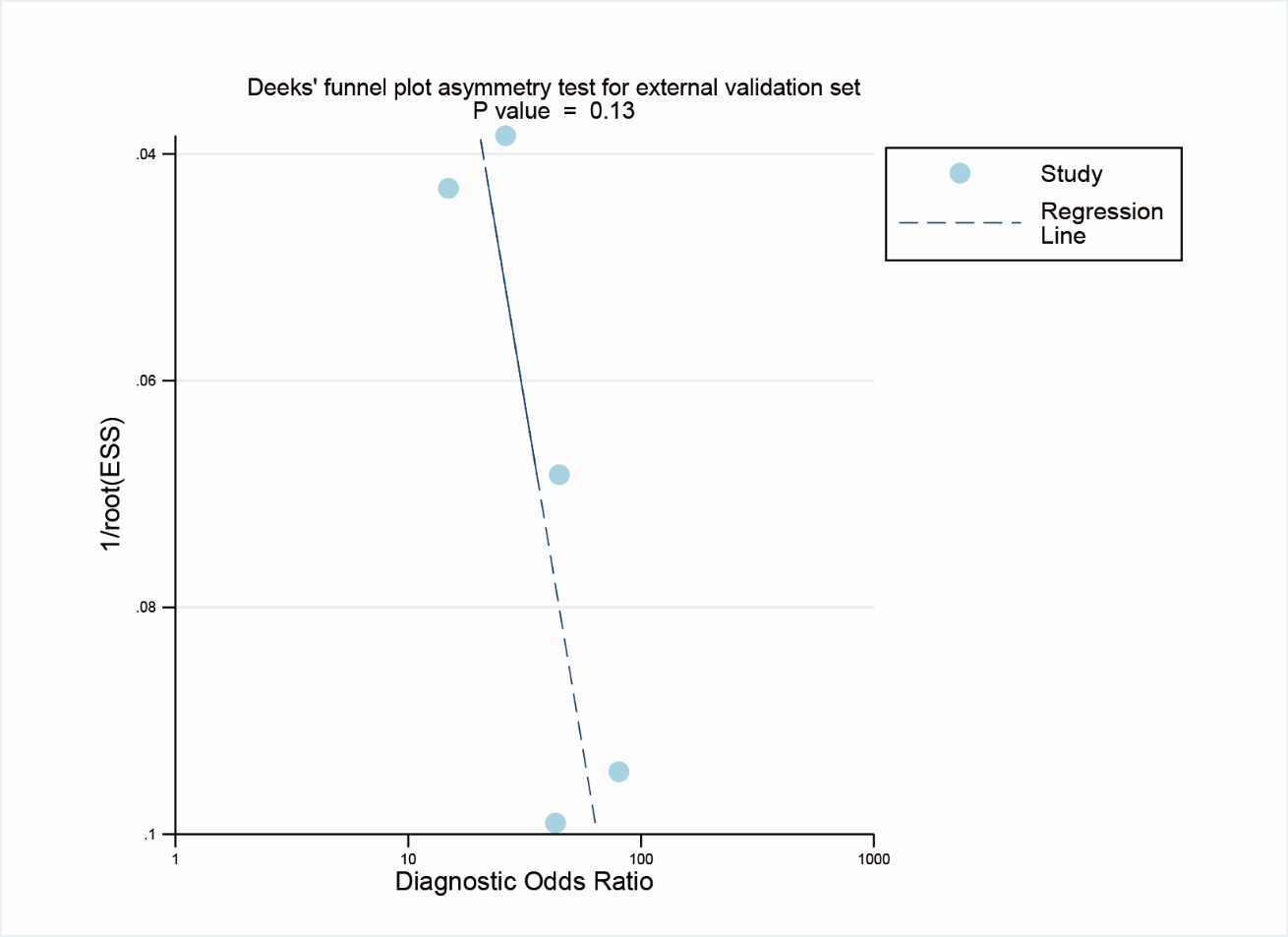

Supplement: Supplementary file 1 [file DataSheet1.docx]
